# Chitosan-Based Nanoencapsulation of Mānuka Oil for Periodontal Treatment

**DOI:** 10.3390/ijms262010201

**Published:** 2025-10-20

**Authors:** Chen Chen, Warwick J. Duncan, Natalie J. Hughes-Medlicott, Ghsaq Alhamdani, Dawn E. Coates

**Affiliations:** 1 Sir John Walsh Research Institute, Faculty of Dentistry, University of Otago, Dunedin 9016, New Zealand; chen.chen@postgrad.otago.ac.nz (C.C.); alhgh812@student.otago.ac.nz (G.A.); dawn.coates@otago.ac.nz (D.E.C.); 2 School of Pharmacy, University of Otago, Dunedin 9016, New Zealand; natalie.hughes@otago.ac.nz

**Keywords:** nanoencapsulation, mānuka oil, periodontal disease, antimicrobial

## Abstract

Periodontal diseases are local bacterial infections that cause inflammation in periodontal (gum) tissues, significantly impacting a patient’s quality of life. Current clinical treatment, such as scaling and root planing combined with antibiotics, shows drawbacks, including antibiotic resistance. The potential of plant-derived bioactives with antimicrobial properties has led to growing interest in the biomedical field. Mānuka oil, an essential oil derived from the Leptospermum scoparium plant, is a potential candidate with known antibacterial and anti-inflammatory properties. Formulation of natural oils for delivery, sustained release, and substantivity in the oral environment is challenging. The integration of nanoencapsulation technology offers the potential to prolong the release time, as well as enhance biocompatibility and address the current therapeutic requirements. Microfluidics enables precise nanoparticle synthesis, while chitosan, due to its antimicrobial activity, muco-adhesion, and biocompatibility, represents a promising encapsulation polymer. This paper aims to review antimicrobial essential oils and nanoencapsulation methods, highlighting the application of microfluidics in developing mānuka oil-loaded chitosan nanoparticles for local delivery in periodontal treatment.

## 1. Introduction

Periodontal disease is an infection and inflammation condition in the oral cavity [1]. According to the World Health Organization report in 2024, periodontal diseases affect approximately 19% of the world’s adult population [2]. Current research confirms that periodontal disease originates from dental bacterial plaque formation. The toxins and enzymes produced by bacteria activate the host’s immune response and damage the periodontal structures [3]. For a susceptible host, oral bacteria coaggregate through adhesin-receptor interactions, leading to the formation of mature dental plaque [4].

**Figure 1:**
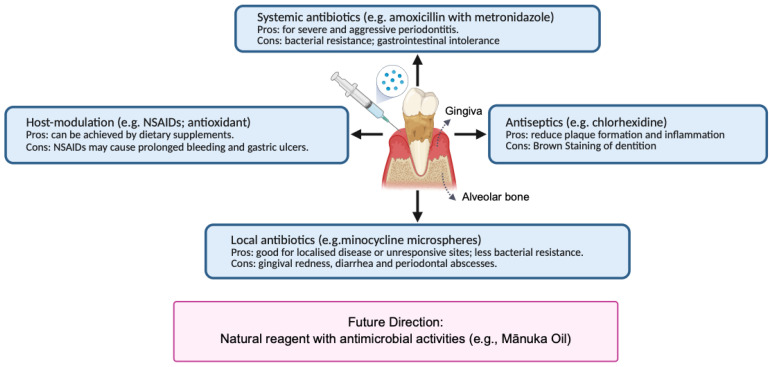
Adjunctive therapies for scaling and root planing to treat periodontal disease (Created with BioRender.com).

The main goals of periodontal treatments are to halt disease progression, minimize symptoms, regenerate lost tissues, preserve healthy tissues, and achieve long-term periodontal maintenance [5]. Scaling and root planing (SRP) is an essential and non-surgical treatment involving the removal of bacterial deposits or calculus using hand or ultrasonic instruments and the smoothing of root surfaces. Studies have confirmed the effectiveness of SRP in reducing inflammation and bacterial burden in patients with periodontitis [6]. However, SRP treatment alone can have limitations, including residual biofilm and calculus; difficulty accessing complex areas; limited effectiveness in eliminating bacteria from soft tissues; and possible side effects, such as dentin hypersensitivity [7,8,9,10,11,12]. Adjunctive therapies may be integrated to enhance the effectiveness of non-surgical treatment [13]. Currently, various adjunctive therapies for SRP have been used in clinical treatment, including the administration of antiseptics, systemic antibiotics, local antibiotics, and host-modulating agents (Figure 1) [5,14]. Due to growing concerns about drug resistance and other limitations, alternative natural bioactives with antimicrobial activities have attracted significant attention in treating bacterial infection, including periodontal disease [15]. This review explores the antimicrobial properties and safety of plant-based essential oils, in particular, mānuka oil, and investigates the potential application of nano-delivery systems in the periodontal pocket as an adjunctive approach to SRP in treating periodontal disease.

Nanoencapsulation of the essential oil enables local drug delivery to the target lesion. This approach offers advantages, including reduced peak concentrations, controlled and sustained release, and improved therapeutic effects [16]. In recent years, microfluidics technology has gained significant attention in nanoparticle synthesis due to its reproducible and precise process compared to conventional bench-top methods [17,18].

This review first explores the antimicrobial properties and safety of plant-based essential oils, in particular, mānuka oil, and investigates the potential application of nano-delivery systems in the periodontal pocket as an adjunctive approach to SRP in treating periodontal disease. Next, various nanoencapsulation strategies for essential oils are discussed, highlighting the application of microfluidic systems. Finally, different polymers used in essential oil encapsulation are evaluated, with special focus on chitosan for its antimicrobial, muco-adhesive, and biocompatible properties, which make it suitable for the local intra-periodontal pocket delivery.

## 2. Essential Oils as Anti-Inflammatory and Antimicrobial Agents for Periodontal Treatment

Essential oils are complex natural compounds extracted from different plant parts, such as leaves, bark, seeds, and wood. Their antimicrobial properties have been investigated in recent years. Major bioactive constituents for their antimicrobial properties include terpenes, ketones, esters, aldehydes, and sulfides, which disrupt the cell membranes, increase the membrane permeability, impair mitochondrial function, and cause electrolyte leakage [19,20,21,22,23]. Beyond their antimicrobial activities, essential oils also exhibit anti-inflammatory effects, demonstrating their potential application in periodontal treatment [19,24].

### 2.1. Anti-Inflammatory Effects of Essential Oils

The anti-inflammatory effects of essential oils have also been widely studied. Major bioactive compounds and underlying mechanisms are listed in Table 1, showing their potential application in periodontal therapy.

### 2.2. Antimicrobial Properties of Essential Oils Against Oral-Related Bacteria

The antibacterial activity of various essential oils, including mānuka oil (*Leptospermum scoparium*), tea tree oil (*Melaleuca alternifolia*), eucalyptus oil (*Eucalyptus globulus*), rosemary oil (*Rosmarinus officinalis*) and lavender oil (*Lavandula angustifolia*), were assessed by comparing their minimum inhibition concentration (MIC) and minimum bactericidal concentration (MBC) values against oral pathogens (Table 2). Among these, mānuka oil exhibited the lowest MIC and MBC values, indicating its strong antibacterial effects [38,39,40,41,42,43,44]. In addition, mānuka oil effectively inhibited the adhesion of *P. gingivalis* and *Streptococcus mutans* (*S. mutans*) onto the cell surfaces, preventing the formation of dental plaque [39].

Various studies have further investigated the antibacterial activity of mānuka oil against different bacterial strains. Kim et al. [45] reported the MIC values against *S. mutans*, *Streptococcus sanguinis*, *Staphylococcus epidermidis*, and *Escherichia coli* (*E. coli*) were 0.08%, 0.08%, 0.02% and 1.25%, respectively. When combined with Tris-EDTA, mānuka oil showed synergistic antibacterial effects against bacteria isolated from otitis externa (swimmers’ ear). The MIC values of mānuka oil against *Pseudomonas aeruginosa*, *E. coli*, *Klebsiella pneumoniae*, and *Proteus mirabilis* (including the multidrug-resistant) were 8%, 2–4%, 2–8% and 2–4%, respectively. After combining with Tris-EDTA, the corresponding MIC values were significantly reduced to 0.5%, 0.12%, 0.25–1% and 0.25–1% [46].

The antibacterial properties of mānuka oil are attributed to its β-triketones components, which disrupt bacterial cell membranes and alter cell morphology, leading to the lysis of bacterial cells [47]. The antibacterial effects of the purified fractions containing the major β-triketones extracted from mānuka oil have also been investigated. The MIC values for grandiflorone, leptospermone and isoleptospermone against methicillin-resistant *Staphylococcus aureus* (*S. aureus*) were 125 μg/mL (0.0125%), 250 μg/mL (0.025%) and 250 μg/mL (0.025%), respectively [48]. Leptospermone also has demonstrated antibacterial effects against multiple foodborne pathogens, with MIC values: *Salmonella typhimurium* (*S. typhimurium*) (23.6 μg/mL; 0.002%), *Listeria monocytogenes* (41.1 μg/mL; 0.004%), *S. aureus* (53.5 μg/mL; 0.005%) and *Shigella flexneri* (65.3 μg/mL; 0.006%) [49].

In addition to the antibacterial properties, mānuka oil has also been reported to have antifungal, antiviral and anticandidal effects [38,50].

Given its strong antimicrobial and anti-inflammatory properties, mānuka oil is discussed in detail in the following section.

**Table 2:** Antimicrobial activities of essential oils. Minimum inhibition concentration (MIC) (%) and minimum bactericidal concentration (MBC) (%) of essential oils against oral pathogens.

Essential Oil	Antimicrobial Mechanism		Major Active Components	References	
Tea tree	Disrupts cellular homeostasis. Inhibits the respiratory activity. Affects membrane integrity.		Terpinen-4-ol Terpinolene α-Terpineol 1,8-Cineole γ-Terpinene	[26,51]	
Eucalyptus	Damages the bacterial cell wall. Alters physiological function. Disrupts membrane integrity and permeability, leading to leakage of intracellular constituents.		1,8-Cineole Citronellal Endo-borneol α-Terpineol Rosifoliol	[52,53]	
Lavender	Interacts with bacterial membrane-associated proteins and metabolic enzymes. Disrupts cytoplasmic membrane and leads to the leakage of intracellular contents.		Carvacrol Linalool Linalyl acetate	[54,55,56]	
Rosemary	Disrupts bacterial lipid bilayer. Leads to the loss of membrane integrity and cellular material.		α-Terpineol Terpinen-4-ol 1,8-Cineole	[57,58]	
Mānuka	Disrupts bacterial cell membranes. Alters cell morphology. Causes the lysis of bacterial cells.		Leptospermone Isoleptospermone Flavesone Grandiflorone	[38,47]	
**MIC (%)**	**M**ā**nuka**	**Tea Tree**	**Eucalyptus**	**Lavender**	**Rosemary**
*S. mutans*	0.25; 0.08; 0.06	1.0; 1.0; 0.26	1.0; >1.0	>1.0	>1.0
*S. aureus*	0.05; 0.02–0.05; 0.03	0.1	4	3.2	0.03; 0.125
*P. gingivalis*	0.03	0.13; 0.065	0.5	0.5	1.0
*A. actinomycetemcomitans*	0.03	0.5; 0.029	0.5	0.5	0.5
*F. nucleatum*	0.03	0.06; 0.085	0.13	0.25; 0.4	0.5
**References**	[39,45,47,59,60,61]	[39,59,62,63]	[39,59,64]	[39,63,65]	[39,66,67]
**MBC (%)**	**M**ā**nuka**	**Tea Tree**	**Eucalyptus**	**Lavender**	**Rosemary**
*S. mutans*	0.25; 2.5	1.0; 1.042	1.0	>1.0	>1.0
*S. aureus*	0.09	0.2	4	6.4	0.1; 0.250
*P. gingivalis*	0.06	0.5; 0.065	0.5	>1.0	1.0
*A. actinomycetemcomitans*	0.13	0.5; 0.052	0.5	>1.0	1.0
*F. nucleatum*	0.03	0.25; 0.169	0.5	>1.0	0.5
**References**	[39,45,60]	[39,62,63]	[39,64]	[39,63]	[39,66,67]

### 2.3. Mānuka Oil

#### 2.3.1. Chemical Compounds of Mānuka Oil

Mānuka (*Leptospermum scoparium*) is a plant native to New Zealand; similar plants are found in Southeast Australia [68]. Since pre-European times, indigenous New Zealand Māori have used mānuka for therapeutic purposes, such as creating infusions to treat fevers and urinary problems and using topical preparations for skin inflammation [69,70]. Mānuka oil is the essential oil extracted from the foliage, bark, and seeds of Leptospermum scoparium plants by steam distillation [71,72]. Gas chromatography–mass spectrometry (GC-MS) results identified more than 100 compounds in mānuka oil, including β-triketones (e.g., leptospermone, isoleptospermone, flavesone, and grandiflorone), calamenene, cineole, and cadinene [38]. The structures of major β-triketones are listed in Table 3. The percentage of chemical components in mānuka oil varies depending on the geographical location. In Australia, *Leptospermum scoparium* oil is rich in monoterpenes but contains low or no triketones. In New Zealand, different regions show different chemotypes: the North Island is high in α-pinene, the Eastern Cape is high in β-triketones, and other regions are high in sesquiterpenes. Among these, oils from the East Cape chemotype exhibit the strongest antimicrobial activity [44,71].

#### 2.3.2. Biocompatibility, Safety, and Efficacy: In Vitro, In Vivo, and Clinical Trial

The biocompatibility and therapeutic effects of mānuka oil have been assessed by in vitro studies with various cell types, by in vivo animal models and in clinical trials (Table 4). In vitro cell experiments demonstrated that mānuka oil ranging from 0.1% to 10% did not show toxic effects on THP-1 cells over 48 h [70]. However, treatment with 500 μg/mL (0.05%) mānuka oil significantly reduced cell viability by 25% in normal human fibroblast cells (CUA-4) and 56% in fibrosarcoma cells (HT-1080) over 24 h [73]. When investigating African green monkey kidney cells (RC-37 cells), the concentration of mānuka oil with non-toxic effects was 0.003% (28.8 μg/mL), while the concentration causing a 50% decrease in cell viability (TC50) was 0.004% (38.4 μg/mL) [74]. The higher safety threshold in THP-1 cells compared with HT-1080 and CUA-4 cells may be attributed to their low proliferation rate, which reduces cellular susceptibility to mānuka oil [75,76].

In an in vivo study with hairless mice, the topical application of 10% mānuka oil (diluted in ethanol) effectively reduced UV-induced cutaneous photoaging, including suppressing epidermal hyperplasia and wrinkle formation in the skin [69]. Incorporating 0.5% mānuka oil into TiO_2_ nanotubes showed excellent bone-healing properties, with significantly improved bone mineral density in a rabbit distal femoral bone defect model, compared with the control [45].

The safety and therapeutic effects of mānuka oil have been further validated in human clinical trials. In one study, participants aged 18 years and older used a mouth rinse with 0.33% (*v*/*v*) mānuka oil twice daily for 30 s over 12 weeks. During the trial, blood tests were performed to ensure safety. No significant differences were observed in the gingival index and plaque index between mānuka oil mouth rinse and placebo group over 12 weeks; however, such results may be attributed to the small sample size of the study. Of note, there were no changes in complete blood counts or comprehensive metabolic blood values, supporting the safety of the tested mouth rinse [77]. Another clinical trial demonstrated that gargling with small volumes of mānuka oil (two drops per time-point for 3 to 5 times per day) for a week had positive effects on radiation-induced mucositis [78].

The European Commission (EC) Regulation reported that MELORA™ Mānuka oil, containing a high percentage of β-triketones, showed a lethal dose to 50% (LD_50_) at 1061 mg/kg via the oral route and LD_50_ over 2000 mg/kg via dermal application. The Campo™ Mānuka Oil formulation was classified as non-irritating based on findings from in vivo animal studies and clinical trials conducted on healthy human participants [50,79]. However, the cytotoxicity and biocompatibility of the pure β-triketone have not been investigated.

## 3. Essential Oil Encapsulation with Nano-Techniques

### 3.1. Non-Encapsulation vs. Encapsulation of Essential Oils

Essential oils are volatile, have a strong aroma, and are unstable, as they are degraded by oxygen, heat, and light. Due to these limitations, it is beneficial to encapsulate the oils within a carrier (a drug delivery system) [80,81]. Other advantages of encapsulation include reduction of peak concentrations, controlled release, prolonged duration of action, and enhanced biological effects [16]. Various encapsulation techniques of essential oils have been explored in recent years, with the most common methods being microencapsulation and nanoencapsulation [82]. Microencapsulation effectively protects and controls the release of loaded compounds, extends shelf life, and masks undesirable flavors. In comparison, nanoencapsulation carriers with a size between 10 and 1000 nm offer additional advantages, such as higher surface area, precise targeting, and enhanced intracellular uptake [82,83].

### 3.2. Nanoencapsulation of Essential Oils

In recent years, nano-scale materials have been widely applied in the biomedical field for disease diagnosis, therapy, and targeted drug delivery [84,85]. The term “nano” has been defined differently. The conventional definition of nanomaterials refers to “materials with at least one primary dimension ranging from 1 to 100 nm” [86,87]. However, considering that nanomaterials with the size of a hundred to several hundred nm can easily enter cells, the size of nanomaterials, especially three-dimensional or bulk nanomaterials, has been defined as <1000 nm [88,89]. Various nano-formulations, including nanotubes, nanofibers, and nanoparticles, have been developed for the encapsulation of essential oils. Table 5 presents an overview of the nanocarriers used for essential oil encapsulation in oral applications. However, the nanoencapsulation of mānuka oil remains underexplored, with only nanotube and nanoemulsion formulations reported to date, as discussed in Section 3.2.1 and Section 4.1.

#### 3.2.1. Nanotubes

Nanotubes synthesized with different materials have been applied to deliver essential oils due to their unique properties, including large surface area, low toxicity, and tubular structure [98]. Studies have reported using halloysite nanotubes to deliver various essential oils, including oregano essential oil, clove essential oil, thyme essential oil, and peppermint essential oil [99,100,101]. For example, oregano essential oil was encapsulated in halloysite nanotubes, which exhibited an average length of 788 nm and a diameter of 115 nm. These nanotubes, with an encapsulation efficiency of 15.8%, enabled a prolonged release of the oil over 12 h in 50% ethanol and exhibited strong antimicrobial and antioxidation properties [98]. Kim et al. [45] developed mānuka oil-loaded titanium dioxide (TiO_2_) nanotubes on a Ti surface with 11.9 ± 2.4 nm in thickness, 88.3 ± 4.8 nm in diameter, and 600 nm in height. The concentration of mānuka oil ranged from 0.02% to <1%, with the nanotubes exhibiting strong antibacterial activity while maintaining good compatibility. In vivo experiments utilizing a rabbit femoral defect model confirmed that nanotubes with 0.5% mānuka oil demonstrated high biological activity and potential for improving bone regeneration.

#### 3.2.2. Nanofibers

Electrospun nanofibers are another option for essential oil encapsulation due to their high surface-to-volume ratio, porous structure, adaptable surface functionalization, stiffness, and resistance to traction [102,103]. Once the nanofibers are placed in an aqueous environment, such as the oral cavity, the encapsulated active compounds are released as the nanofibers degrade [66]. Dadras et al. [104] prepared nanofibers using silk fibroin and gelation to deliver thyme essential oil with an average diameter of 248.37 nm, a porosity of 87.57%, and a loading efficiency of 3.41%. These nanofibers demonstrated sustained release of oil in PBS over 48 h, excellent biocompatibility, and effective antibacterial properties against *S. aureus* and *K. pneumoniae*. Similarly, lemon balm and dill essential oils loaded into collagen hydrolysate–chitosan nanofibers with a mean dimension of 120 nm exhibited strong antimicrobial activities against *S. aureus*, *E. coli*, *Enterococcus faecalis*, *Candida albicans*, *S. typhimurium*, and *Aspergillus brasiliensis*. The biocompatibility and safety of these formulations were validated using a white Swiss mice model with subcutaneous implantation, and follow-on hematological, biochemical, and immunological analyses [105]. Other essential oils derived from lavender, thyme, clove, and peppermint have also been successfully encapsulated into nanofibers for pharmaceutical or biomedical applications [106].

However, the challenges of using nanofibers to deliver active compounds include low yield, the need for high operating voltages during electrospinning, the requirement to modify solution conductivity with oil incorporation, and the need to maintain the stability of the oil during material deposition [102].

#### 3.2.3. Nanoparticles

**Figure 2:**
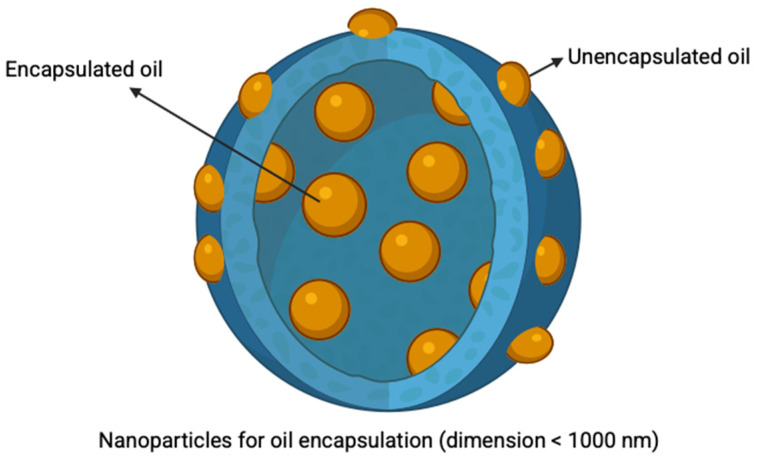
Schematic diagram of a nanosphere with the encapsulation of essential oil (Created with BioRender.com).

Nanoparticles (NPs) ranging in size from 10 to 1000 nm have gained considerable attention in recent years, with a preferred size of <200 nm for nanomedical applications [107]. Their nano-scale size leads to a high surface-area-to-volume ratio that enhances cellular interaction, nanocarrier degradation, and controlled release of encapsulated compounds [108]. The primary uptake mechanism for larger particles of 250–3000 nm is phagocytosis, whereas smaller NPs (<200 nm) are taken up through other alternative routes, such as endocytosis [109,110]. Advantages of NP application include enhanced drug solubility, improved substantivity, controlled release, facilitated co-delivery of multiple therapeutic agents, and improved therapeutic efficacy [111,112,113]. Various methods have been explored to encapsulate essential oils into NPs (Figure 2 and Table 6).

## 4. Methods for Nanoparticle Synthesis for Oil Encapsulation

### 4.1. Emulsification

**Figure 3:**
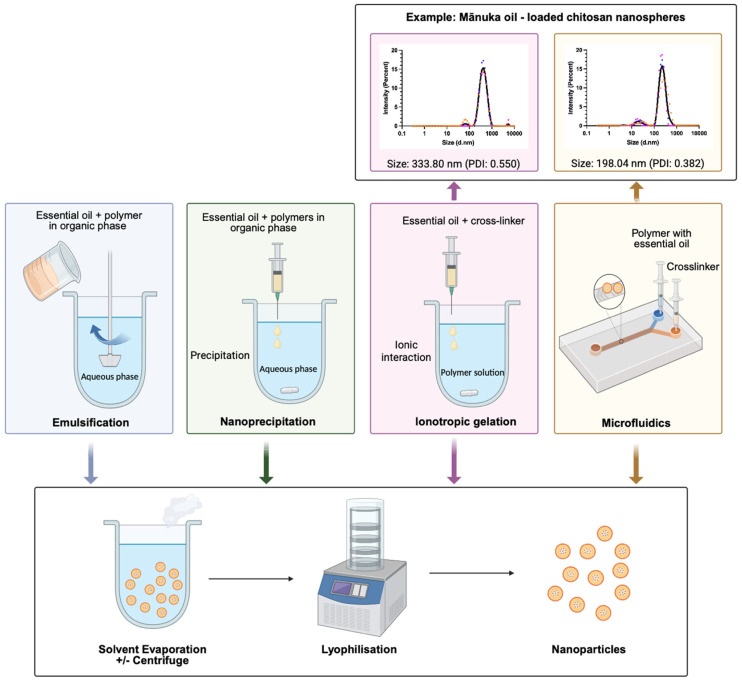
Nanoparticle synthesis for oil encapsulation through different methods, including emulsification, nanoprecipitation, ionotropic gelation, and microfluidics. DLS results represent the essential oil-loaded nanospheres synthesized via ionotropic gelation and microfluidics methods, as reported in our unpublished work. Dots in blue, orange and pink represented 3 times replication, and the black line represented their mean. (Created with BioRender.com).

The synthesis of essential oil-loaded NPs using emulsification methods involves two phases: the organic phase and the aqueous phase. The organic phase involves dissolving the essential oil and polymer (e.g., polycaprolactone) into the organic solvent (e.g., ethyl acetate), which is then emulsified into the aqueous phase, which contains a surfactant (e.g., Tween 80). Nanodroplets are formed through high-speed homogenization or ultrasonication steps (Figure 3) [125]. After the evaporation of solvent, the NPs are washed and collected [114,126]. Various essential oils have been encapsulated into NPs through emulsification methods. Clove essential oil-loaded chitosan NPs with improved antifungal properties were synthesized through emulsion methods and further solidified via tripolyphosphate (TPP) gelation. Depending on the different chitosan-to-oil ratio (*w*/*w*), the particle sizes ranged between 200 and 1200 nm, with the encapsulation efficiency ranging from 31% to 45% [127]. Liang et al. [115] reported that the antibacterial peppermint essential oil-loaded starch nanoemulsions exhibited particle sizes < 200 nm and maintained high stability over 30 days. Another study reported that nanoemulsions containing various essential oils (cinnamon, thyme, mānuka, and tea tree oil) with an average size of 50–100 nm (PDI: 0.428) were prepared using the emulsification method. Surprisingly, the antifungal concentration required for the pure oils (0.5 to 2.5%) was reduced to 0.5% in the nanoemulsion formulation [128].

However, the major drawbacks of the emulsification method include the use of organic reagents, residual surfactants, and aggregation of NPs [129,130,131].

### 4.2. Nanoprecipitation

NP formation through nanoprecipitation relies on interfacial deposition after solvent displacement [132]. The polymer and essential oil are dissolved together in an organic phase and added into the aqueous phase with surfactant under continuous stirring (Figure 3) [125]. After the evaporation of the organic solvent, the NPs are collected via ultracentrifugation or freeze drying [126]. The average size of NPs obtained through this method ranges from 170 to 900 nm, and mixing in small volumes leads to a narrower size distribution [118,125]. Other factors, such as volume ratio, polymer concentration, mixing speed, and choice of the solvent, also affect the particle quality [133]. Liakos et al. [117] encapsulated peppermint, cinnamon, and lemongrass essential oils into cellulose acetate nanocapsules with diameters of 180, 150, and 200 nm (PDI: 0.09 to 0.27), respectively, using the precipitation method. All of these nanocapsules showed low cytotoxicity on amniotic fluid stem cells and strong antimicrobial properties not only against single bacterial cultures but also against biofilms. In other research, Origanum vulgare essential oil was loaded into polycaprolactone (PCL) NPs with an average size between 180 and 230 nm (PDI: 0.130 to 0.180) and an encapsulation efficiency ranging from 50 to 90% [119,120].

Compared with emulsification, this nanoprecipitation method is easy to operate and enables fine NP formation with a narrow particle size distribution. However, there is currently no method to precisely control the particle size and eliminate batch-to-batch variability [133,134].

### 4.3. Ionotropic Gelation

The ionotropic gelation method for NP synthesis is favored for its simple experimental procedure and mild reaction conditions. It relies on the ionic interaction between the polyelectrolytes (e.g., chitosan, hyaluronic acid, and alginate) and the cross-linker with counterions (Figure 3) [135,136]. Various types of essential oils have been encapsulated in NPs synthesized via ionic gelation. Saffron essential oil has been widely used in the food industry for its antimicrobial and antioxidation properties. To enhance its stability against oxidation, saffron essential oil-encapsulated nanoparticles were synthesized through the ionic interactions between chitosan and Arabic gum, a polyanion biopolymer. The optimized formulation of saffron essential oil-encapsulated nanoparticles has a size of 16.22 nm (PDI: 0.4948) and an encapsulation efficiency of 86.4% [122]. Neem oil, the essential oil extracted from Neem (Azadirachta indica) seed, with antibacterial, antifungal, and insecticidal properties, was encapsulated in spherical chitosan–TPP NPs via ionic gelation. The nanospheres had an average size of 132.6 to 196.3 nm (PDI: 0.17 to 0.25) and a loading capacity of 21.90 to 24.03% [121].

### 4.4. Microfluidics

In recent years, microfluidics platforms have become a popular method for synthesizing NPs, due to the small reagent volumes required, precise experimental conditions, cost-effectiveness, simple operation, and short reaction time [17,18]. The diameter and particle size distribution can be tuned by modifying various parameters, such as total flow rate, total flow rate ratio, reagent concentration and pH, and reaction temperature. Compared to traditional NP synthesis methods performed on the bench, these precise adjustments ensure consistent synthesis conditions between different batches, facilitate scalability, and enable the reproducible manipulation [17,137]. These features provide a promising strategy to overcome major challenges in the clinical translation of nanomedicine [137,138].

NP synthesis using microfluidics devices can be divided into two types based on the different mixing flow types, which are single-phase flow and multiple-phase droplet flow. Single-phase flow systems are especially effective for self-assembly NP production, where NP is formed through diffusion-based laminar flow in the channel. The steady fluid flow contributes to the reproducible and controllable environment for reaction, leading to a narrow particle size distribution [139]. Drawbacks such as clogging issues, long diffusion time and cross-contamination of the single-phase flow systems are countered using droplet-based microfluidic, which facilitates the synthesis of NPs in the micro-droplet reactors in the main channel [18,140]. The device has multiple patterns, including T-junction, Y-junction, and S-channel. The fluid flow is transported through side junctions in the form of droplets and merges into the main channel, where NP production occurs [18,141] (Figure 3).

The synthesis of essential oil-encapsulated NPs using microfluidics has been reported in a limited number of studies. Helal et al. [123] used the microfluidic system to fabricate spherical chitosan-coated PLGA NPs encapsulating three types of essential oil (linalool, geraniol, and eugenol), with sizes ranging from 200 to 600 nm and encapsulation efficiencies of 79.68, 71.34, and 95.14%, respectively. The NPs showed burst release of the oil within the first 8 h and sustained release over 120 h. The encapsulation of essential oil in NPs significantly reduced the cytotoxicity compared with free oil over 48 h.

## 5. Polymers Used in Nanoparticle Synthesis to Deliver Essential Oils

Various polymers have been used for NP synthesis to deliver essential oils, as listed in Table 7. Dupuis et al. quantified the publications using various agents for NP synthesis. By 2020, the number of publications had increased 3.5 times compared with 2010, and among them, chitosan has become increasingly popular as a nanocarrier agent, accounting for 42.8% of studies, followed by lipid (27.3%), cellulose (12.6%), and PLGA (12.15%) [142]. The properties of chitosan are discussed in detail below.

## 6. Chitosan

### 6.1. Chitosan Characteristics

**Figure 4:**
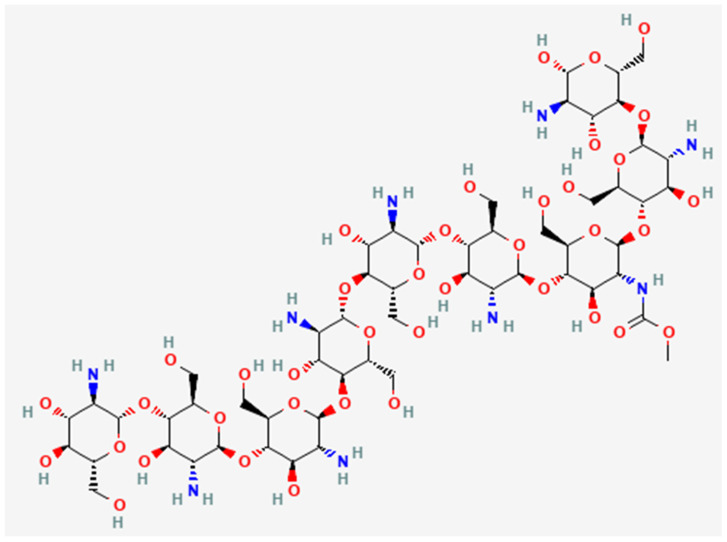
Chemical structure of chitosan [158].

Chitosan is a chitin derivative obtained through deacetylation under alkaline conditions. It is composed of β-(1→4)-linked D-glucosamine (deacetylated unit) and N-acetyl-D-glucosamine (acetylated unit) [155,156] (Figure 4). Compared with chitin, which is insoluble in water, organic solvents, and mild acidic or basic solutions, chitosan shows high solubility in mild acidic solvents, and its solubility depends on the molecular weight, as well as the acetylation degree [156,157]. Other properties, including high biocompatibility, muco-adhesion, and antimicrobial effects, make chitosan a favorable material for intra-pocket local drug delivery in periodontal therapy. A detailed discussion about these properties is presented in the following sections.

### 6.2. Biocompatibility

Chitosan is a non-toxic biocompatible polymer that has been approved by the United States Food and Drug Administration (FDA) as a food additive and wound-dressing material [159,160]. Its biocompatibility has been assessed in various studies through in vitro cell experiments and in vivo animal models. Richardson et al. [161] reported that chitosan exhibited a half-maximal inhibitory concentration (IC_50_) below 1 mg/mL against L132 human embryonic lung cells and CCRF-CEM human lymphoblastic leukemia cells. When chitosan NPs were cross-linked with TPP, cytotoxic effects were not observed until a concentration of 5 mg/mL was reached on human gingival fibroblasts [162]. Chitosan–TPP NPs also exhibited lower cytotoxicity compared to free chitosan solution when tested on the TR146 cell line [163]. Chitosan–mucin NPs demonstrated no cytotoxicity on the HT-29 human colorectal adenocarcinoma cell line at a concentration of 500 μg/mL, and their safety was further validated in vivo through oral administration in Wistar rats [164]. The toxicity and safety of chitosan have been evaluated in rats, rabbits, and dogs by different routes, including oral administration (e.g., gavage and dietary) and injection. In rats, no toxicity was observed with gavage dose 2000 mg/kg/day, and dietary dose 3000 mg/kg/day for 3 months [165]. Rabbits were given chitosan through intravenous injection at 4.5 mg/kg/day over 10 days and orally at 800 mg/kg/day for 34 weeks without causing abnormal changes [166]. Subcutaneous injection of chitosan to mongrel dogs began to cause transient appetite loss and anorexia at 30 mg/kg/day, an increased white blood cell counts at 50 mg/kg/day, and mortality at above 150 mg/kg [166,167,168].

The safety of chitosan’s local application in the oral cavity has also been investigated. In a high-fat-diet-induced rat periodontitis model, weekly topical application of chitosan-based injectable hydrogel (1.71% *w*/*v* chitosan, 60 μL) for 2 weeks, as an adjunct to SRP, reduced inflammation and promoted periodontal healing compared with SRP alone [169]. Clinical trials also support its potential in periodontal treatment. Local administration of chitosan gel (1% *w*/*w*) after SRP twice per week for 24 weeks led to a significant reduction in probing depth (from 0.94 mm to 1.21 mm) compared to SRP alone in patients with chronic periodontitis [170]. Another study demonstrated that application of chitosan gel (1% *w*/*v*) either alone or in combination with demineralized bone matrix/collagen membrane following flap surgery improved bone fill, as confirmed by radiographic analysis at 3 and 6 months [171].

### 6.3. Antimicrobial Properties

The antimicrobial properties of chitosan have been explored, and the most widely accepted mechanism is that the positively charged NH_3_^+^ competes with the Ca^2+^ channel on the negatively charged bacterial cell membrane, which changes membrane permeability and leads to the leakage of intracellular compounds [172]. Antibacterial activity of chitosan with different molecular weights (from 28 to 1671 kDa) has been investigated against various bacteria. The MIC values ranged from 0.05% to >0.1% depending on the bacterial species and chitosan molecular weights (Table 8) [173].

Antibacterial activity of chitosan against oral pathogens was further reported by Costa et al. [174], showing that MIC values of low- and high-molecular-weight chitosan ranged from 1 to 5 mg/mL (MBC: ranging from 3 to 7 mg/mL) against *P. gingivalis*, *Tannerella forsythensis* (*T. forsythensis*), *Prevotella buccae* (*P. buccae*), *A. actinomycetemcomitans*, *Prevotella intermedia* (*P. intermedia*), and *S. mutans* (Table 8).

### 6.4. Muco-Adhesive Properties

Muco-adhesive biomaterials have attracted significant attention in local drug delivery. Chitosan has strong muco-adhesive properties through several mechanisms, including (1) formation of intermolecular bonds—the amine groups of chitosan form hydrogen bonds with the amino acids of the mucosa; (2) electrostatic interaction—the positively charged amine groups of chitosan interact with the negatively charged sialic acid residues on mucin glycoproteins; and (3) chitosan penetrates the pores on the tissue surface [175]. The muco-adhesion of chitosan particles is greatly affected by its cross-linking method. Chitosan spheres prepared via emulsification plus the ionotropic gelation method or pure ionotropic gelation method showed better muco-adhesion compared to those produced through chemical or thermal cross-linking methods. This may be because the spheres prepared by ionic gelation have higher charge values, leading to a stronger interaction between chitosan and mucin [130,176].

A chitosan-based gel formulation has also been developed as a muco-adhesive delivery system for nystatin to treat oral mucositis. In vivo studies on hamsters and healthy volunteers showed that the application of the nystatin-encapsulated gel prolonged drug delivery and retention in the oral cavity compared to nystatin suspension [177]. Similar results were observed in another study with the Wistar rat periodontitis model, showing that the atorvastatin-encapsulated chitosan gel exhibited strong bio-adhesive properties and sustained drug retention at the target site for treating periodontitis by reducing the levels of pro-inflammatory cytokines [178].

## 7. Conclusions and Future Perspectives

This review highlighted the anti-inflammation effects and antimicrobial properties of naturally derived essential oils, with mānuka oil as a key example for periodontal application. In recent years, there has been growing interest in the possibility of using natural antibacterial products due to the rising concern about antibiotic resistance. Nano-delivery systems for mānuka oil provide prolonged release, enhanced biocompatibility, and improved therapeutic efficacy. Various synthesis methods for the essential oil-loaded nanoparticles were discussed in this review, with an emphasis on the application of the microfluidics platform, which enables a controllable, precise, and reproducible production procedure. Delivering plant-derived mānuka oil in chitosan nanospheres using microfluidics holds great promise in developing novel, topically active, sustained-release medicaments for periodontal treatment; however, this approach remains relatively unexplored. Further work should address challenges, including long-term toxicity evaluation, scalability to clinical-grade production, and in vivo assessment in large animal models of periodontitis.

Looking ahead, the treatment of periodontal disease is expected to involve multiple disciplines, bringing together biologists, bioengineers, and dental clinicians to produce natural antimicrobial and regenerative materials for patient care.

## Figures and Tables

**Table 1:** Anti-inflammatory effects of essential oils.

Essential Oil	Anti-Inflammation Mechanism	Major Active Components	References
Tea tree	Inhibit LPS-induced production of TNF-α, IL-1β, IL-8 and IL-10.	Terpinen-4-ol α-Terpineol 1,8-Cineole	[25,26]
Eucalyptus	Inhibit LPS-induced inflammation by reducing MAPK and NF-κB pathways.	1,8-Cineole α-Pinene	[27,28]
Lavender	Decrease production of inflammatory mediator, including TNF-α, IL-1β, NO and PGE2; block NF-κB pathway.	Linalool Linalyl acetate	[29,30]
Clove	Alter NF-κB pathway; reduce production of IL-6 and COX-2.	Eugenol	[31,32]
Peppermint	Activate AMPK, ULK1 and NRF2 pathway; down-regulate MAPKs and NF-κB pathways; suppress oxidative stress and production of NO.	Menthol menthone	[33,34,35]
Mānuka	Inhibit LPS-induced production of TNF-α, IL-1β, IL-6 and NO; Suppress MAPKs and NF-κB pathways.	α-Pinene	[36,37]

**Table 3:** Two- and three-dimensional structures of major β-triketones.

**β-Triketones**		**2D Structure**	**3D Structure**
Leptospermone [40]	C_15_H_22_O_4_	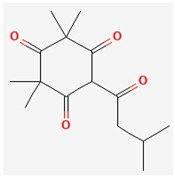	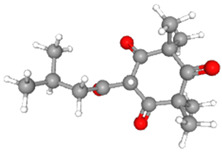
Isoleptospermone [41]	C_15_H_22_O_4_	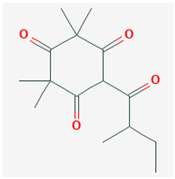	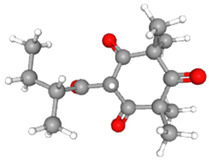
Flavesone [42]	C_14_H_20_O_4_	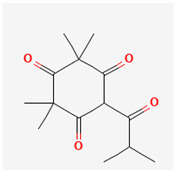	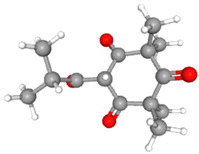
Grandiflorone [43]	C_19_H_22_O_4_	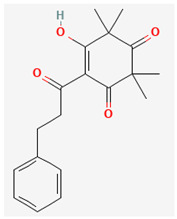	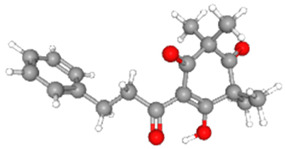

**Table 4:** Biocompatibility, safety, and efficacy of mānuka oil.

In Vitro Cell Experiment		Mānuka Oil Concentration	Effect
Normal human fibroblast cells (CUA-4)		0.05%	Reduced 25% cell viability over 24 h [73].
Fibrosarcoma cells (HT-1080)		0.05%	Reduced 56% cell viability over 24 h [73].
THP-1 cells		0.1% to 10%	No toxic effect over 48 h [70].
African green monkey kidney cells (RC-37 cells)		0.003%	No toxic effect [74].
		0.004%	50% decrease in cell viability [74].

**In Vivo Animal Experiment**		**Mānuka Oil Concentration**	**Effect**
Rabbit	Femoral bone defect model	0.5%	Improved bone healing [45].
Hairless mice	Topical application	10%	Reduced UV-induced cutaneous photoaging [69].

**Human Clinical Trial**		**Mānuka Oil Concentration**	**Effect**
Gargling		2 drops diluted in water per time, 3–5 times per day	Improvement in treating radiation-induced mucositis [48].
Mouth rinse		0.33%	No side effects as reported from hematological test [77].

**Table 5:** Nanoencapsulation of essential oil for oral application.

Essential Oils	Nanocarriers	Size (nm)	Encapsulation Efficiency (%)	Release Kinetics (h)	Muco-Adhesion or Penetration	Antimicrobial Activity/ Therapeutic Efficacy	Oral Application
Oregano and Lemongrass oil [90]	Chitosan nanoparticle	100–200	NA	6 to 24 (distilled water).	NA	In vitro antimicrobial: *C. albicans*	Tissue conditioner
Cinnamon and clove oil [91]	Carbopol 640 Nanoemulgel	152	95.78 (Cinnamon) 96.45 (Clove)	2 to 24 (Franz diffusion cell with PBS, pH 6.8).	Model: in vitro goat buccal mucosa. Adhesive strength: 42.87 N/cm^2^ Drug permeation: 54.21% (cinnamon) and 52.72% (clove) over 24 h.	In vitro antimicrobial: *P. aeruginosa* *B. chungangensis* *S. epidermidis* *S. aureus* *C. albicans*	Oral bacterial infections
Clove oil [92]	Nanolipid	190–220	98.95	8 to 72 (buffer, no specific information).	Model: in vitro dog canine gingival tissue. Penetration: reaching 100 µm depth within 45 min.	In vitro antimicrobial: *E. coli* *Salmonella* spp. *K. Pneumoniae* *B. pyogenes*	Periodontal therapy
Curcuma caesia oil [93]	PLGA–lecithin nanocarrier	51.28	86.4	69.26% over 24 h (PBS, pH 6.8).	Model: in vitro goat buccal mucosa Permeation: 42.79% oil release over 24 h (incorporated in Carbopol 934 P gel).	In vitro antimicrobial: *E. coli* *S. aureus* *L. acidophilus* *P. aeruginosa* *B. subtilis*	Periodontal infections
					Model: in vivo ligature-induced rat periodontitis model (incorporate in Carbopol 934 P gel). Application: topical application with cotton swab. Plasma pharmacokinetic: mean residence time was 4.3 h.	In vivo therapeutic efficacy: reduce inflammatory cells and cellular debris.	
Cinnamon oil with grapeseed extract [94]	Chitosan/carrageenan nanoparticle	357	NA	NA	Model: in vitro mucin adsorption method. Mucin adsorption: 20–40%.	In vitro antimicrobial: *S. mutans* *S. sobrinus*	Mouth rinse
Cinnamon oil [95]	Nanoemulsion	NA	Loading: 5%	NA	NA	In vitro antimicrobial: muti-species oral biofilm on bovine enamel.	Dental caries management
Eucalyptus oil [96]	Nanoemulsion	100–180	NA	NA	NA	In vitro antimicrobial: *S. mutans*	Mouthwash
Clove oil with baicalin [97]	Microemulsion	26.53	93.8	4 h (NaCl 0.9% containing 25% *w*/*v* PEG400)	NA	In vivo therapeutic efficacy (incorporate in P407/P188 gel): reduce inflammatory response and alveolar bone loss.	Periodontal tissue repair

**Table 6:** Different published methods for producing essential oil-loaded nanoparticles.

Methods	Encapsulated Essential Oil	Encapsulation Materials	Size (nm)	PDI	References
Emulsification	Lime	Emulsified in water with Tween 80 as surfactant	21	0.444	[80,114]
			28	0.469	
			60	0.557	
	Peppermint	Emulsified in starch and water	180–230	0.250–0.340	[115]
	Oregano	Zein	>100	NA	[116]
Nanoprecipitation	Peppermint	Cellulose acetate	180	0.09–0.27	[117,118]
	Cinnamon		150		
	Lemongrass		200		
	Origanum vulgare	Polycaprolactone (PCL)	180–230	0.130–0.180	[119,120]
Ionic gelation	Neem	Chitosan	133–175	0.170–0.260	[121]
	Saffron	Chitosan–Arabic gum complex	16–24	0.495–0.607	[122]
Microfluidics	Eugenol, linalool, and geraniol	Chitosan coated PLGA	NA	NA	[123]
	Frankincense	Chitosan with TPP	118	<0.28	[124]

**Table 7:** Polymers for nanoparticle synthesis to deliver essential oils.

Polymers	Example	Advantages	Limitations
Cellulose	Encapsulated peppermint, cinnamon, and lemongrass essential oils into cellulose acetate-based spherical nanocapsules [117].	Non-toxic. Biocompatible. Biodegradable. Cost-effective [143,144].	Polymer without intrinsic antimicrobial properties [145].
Zein	Encapsulated eugenol and garlic essential oils into zein NPs [146].	Better prevention against oxidation and degradation of the encapsulated compounds [147].	Further toxicological studies required [142].
Sodium alginate	Encapsulated lippia sidoides essential oil into alginate–cashew gum NPs [148].	Non-toxic. Biocompatible. Biodegradable. Interacted with agents with different charge [149].	Polymer without antimicrobial properties [145].
PLGA	Encapsulated *Cymbopogon citratus* essential oil with PLGA NPs [150].	Non-toxic. Biocompatible. Biodegradable and easily modified [150].	High production cost. Low encapsulation efficiency [145].
Chitosan	Encapsulated *Cynometra cauliflora* essential oil, curry leaf essential oil, and clove essential oil [151,152,153].	Muco-adhesive. Intrinsic antimicrobial effect. Biodegradable. Biocompatible [152,154].	No major limitations reported [142].

**Table 8:** Minimum inhibitory concentrations (MICs, mg/mL) of low-molecular-weight (LMW) and high-molecular-weight (HMW) chitosan [173,174].

Bacteria		Molecular Weight of Chitosan (kDa)							
		28	107	224	470	624	746	1106	1671
Gram-positive	*S. aureus*	>1.0	-	>0.8	0.8	-	>0.8	>1.0	>1.0
	*Bacillus megaterium*	>0.8	-	>0.5	0.5	-	>0.8	>0.5	>0.8
	*Listeria monocytogenes*	>1.0	-	>0.8	0.8	-	>0.8	>1.0	>1.0
	*Lactobacillus brevis*	>0.8	-	>1.0	1.0	-	>1.0	>0.5	>0.8
Gram-negative	*E. coli*	>1.0	-	>1.0	0.8	-	>0.8	>1.0	>1.0
	*Pseudomonas fluorescens*	>1.0	-	>0.8	0.8	-	>0.8	>1.0	>1.0
	*S. typhimurium*	>1.0	-	>1.0	0.8	-	>1.0	>1.0	>1.0
	*Vibrio parahaemolyticus*	>1.0	-	>1.0	0.8	-	>0.8	>1.0	>1.0
**Oral-Related Bacteria**		**Molecular Weight of Chitosan (kDa)**							
		**28**	**107**	**224**	**470**	**624**	**746**	**1106**	**1671**
Gram-positive	*S. mutans*	-	5	-	-	5	-	-	-
Gram-negative	*P. gingivalis*	-	1	-	-	1	-	-	-
	*T. forsythensis*	-	3	-	-	1	-	-	-
	*P. buccae*	-	1	-	-	3	-	-	-
	*A. actinomycetemcomitans*	-	3	-	-	5	-	-	-
	*P. intermedia*	-	3	-	-	1	-	-	-

-: Not tested.

## Data Availability

No new data were created or analyzed in this study. Data sharing is not applicable to this article.
